# Using a Virtual Patient via an Artificial Intelligence Chatbot to Develop Dental Students’ Diagnostic Skills

**DOI:** 10.3390/ijerph19148735

**Published:** 2022-07-18

**Authors:** Ana Suárez, Alberto Adanero, Víctor Díaz-Flores García, Yolanda Freire, Juan Algar

**Affiliations:** 1 Department of Preclinical Dentistry, School of Biomedical Sciences, Universidad Europea de Madrid, 28670 Madrid, Spain; ana.suarez@universidadeuropea.es (A.S.); victor.diaz-flores@universidadeuropea.es (V.D.-F.G.); yolanda.freire@universidadeuropea.es (Y.F.); 2 Department of Clinical Dentistry, School of Biomedical Sciences, Universidad Europea de Madrid, 28670 Madrid, Spain; juan.algar2@universidadeuropea.es

**Keywords:** artificial intelligent, chatbot, virtual patient, diagnosis, dental students

## Abstract

Knowing how to diagnose effectively and efficiently is a fundamental skill that a good dental professional should acquire. If students perform a greater number of clinical cases, they will improve their performance with patients. In this sense, virtual patients with artificial intelligence offer a controlled, stimulating, and safe environment for students. To assess student satisfaction after interaction with an artificially intelligent chatbot that recreates a virtual patient, a descriptive cross-sectional study was carried out in which a virtual patient was created with artificial intelligence in the form of a chatbot and presented to fourth and fifth year dental students. After several weeks interacting with the AI, they were given a survey to find out their assessment. A total of 193 students participated. A large majority of the students were satisfied with the interaction (mean 4.36), the fifth year students rated the interaction better and showed higher satisfaction values. The students who reached a correct diagnosis rated this technology more positively. Our research suggests that the incorporation of this technology in dental curricula would be positively valued by students and would also ensure their training and adaptation to new technological developments.

## 1. Introduction

Diagnosis is the foundation on which all medical treatments are based. Making a correct, effective, and efficient diagnosis is a fundamental skill that dental students must acquire to be good practitioners. Diagnostic learning in the undergraduate curriculum can be effectively re-enacted through repeated practice of clinical cases with subsequent feedback from faculty, as well as by encouraging self-evaluation to hold students accountable for their deficiencies [1,2].

During undergraduate training it is common to focus on elaborate clinical cases in which trainees must rely on several diagnostic tests before they can make their diagnostic judgment. But it has been questioned whether an extremely detailed anamnesis can be counterproductive if trainees get lost in irrelevant details [3]. In fact, authors such as Bordage [4] urge practice with more focused cases that are based on important discriminative symptoms so that the student can practice with a larger number of clinical cases, a fundamental requirement for acquiring diagnostic competence [2].

In dental education, as a medical discipline, much of the students’ professional development occurs when they begin to interact with patients [5], i.e., when they begin to develop interpersonal communication. However, sometimes patients with good cases, from a teaching point of view, are not available for all students and this causes a limitation in the possibilities of student interaction with a large number of cases [5], which is why in recent years, the use of simulation for the development of students’ psychomotor skills has become standard in dental education because it allows them to follow an appropriate learning curve in a less stressful and controlled environment than in a clinic [6].

Simulation in which interactions with patients are recreated, such as role-plays with teachers, with patient-instructors, or standardized patients, are already commonly used in dental schools [7] and are perceived by students as very positive because of their similarity to their professional practice [8] and also allow increasing the realistic self-assessment of the students [7]. In order to perform these simulations of personal interaction with a standardized patient, a high level of planning and training is required by the organizers [9], which could make it difficult to perform them regularly, as well as the appearance of variables that are not foreseen in the original script that can cause the simulation to fail.

In this sense, virtual patients (VP) are part of the integration of new technologies in patient simulations and could favor a greater practice of clinical cases by students, printing knowledge more effectively [10], facilitating the planning of cases to teachers, and with less budget and infrastructure [11]. With the use of VPs, students can perform learning with greater self-nomination [12]; the learning of a strategic and self-reflective nature with the advantage of the ubiquity that is provided by technology [13].

It is, therefore, an excellent resource as a complement to interaction with real patients [14] when direct contact with the patient is not yet possible [10] due to a lack of preparation of the student or situations such as that which was caused by the COVID-19 pandemic, also allowing the recreation of unusual clinical cases in daily practice [15]. 

In general, VPs are usually well perceived by students because of all the advantages that were previously pointed out [16], but they are not free of limitations such as a disconnect between the available VP programs and the needs of educators [17], or that VPs are usually concentrated on a single pathology while in reality different pathologies can coexist at the same time [18]. Moreover, it should be taken into account that, according to different studies [19,20], students prefer certain features in VP design such as relevance, an adequate level of difficulty, feedback, high interactivity, and above all realism [16]. In this sense, artificial intelligence (AI), defined as that technology that uses machines to mimic intelligent human behavior [21], offers a range of possibilities in the development of VPs due to the ability of AI to allow a computer system to perform perceptual processes that are typical of a human being [22,23,24], offering more realism to the interaction with the VP, in addition to being part of the most promising areas of medicine [25].

In recent years, it has been observed how young people invest less time in learning and more in the use of their cell phone [26]. In this context, chatbots or conversational agents through an instant messaging service are presented in the literature as an application of the emerging field of AI [27] that could attract the attention of students and, therefore, be an interesting alternative in the development of VPs [6,28,29]. 

In relation to education, despite the fact that advances in clinical dentistry have been adapting to digital technological developments that integrate the area of diagnosis and treatment [30,31], it is suggested that there is a need for more research at the academic level on the impact of the use of these digital technologies in clinical practice, with special attention to the ethical issues that may arise as well as the need for dental educators to integrate them into the curriculum [31]. The integration of technology into dental education also makes it possible to implement improvements in patient safety, as it allows practice in scenarios in which the health of a real patient is not compromised [32].

In the specific field of dentistry, some works [6,32,33,34] investigate the use VPs in dentistry, but no studies were found that integrated VPs with AI. For all of the above, the creation and assessment of a VP through an AI chatbot for the development of diagnostic skills of pulp pathology in dental students was proposed as the objective of the present study.

## 2. Materials and Methods

The present descriptive cross-sectional study was approved by the research committee of the Universidad Europea de Madrid (CIPI/22.142).

### 2.1. Participants

Students in the 4th and 5th year of the degree in dentistry at the Universidad Europea de Madrid who were taking practical courses with patients participated in the study. All the students who wished to take part in the study had to sign an informed consent form in which they were informed about the study and were assured that their data would be treated anonymously.

### 2.2. Sample Size

**Figure 1:**
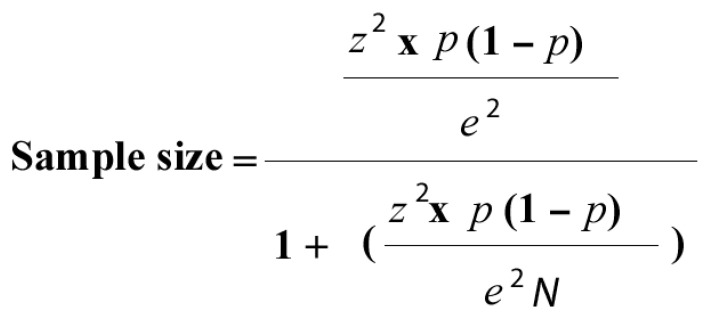
N: population size; z: z-score; e: margin of error (percentage in decimal form).

With a total of 457 students of 4th and 5th year of dentistry enrolled in the subjects with clinical practice at the Universidad Europea de Madrid, the formula that is shown in Figure 1 was applied to calculate the sample size. A confidence percentage of 95% and a margin of error of 6% were taken into account and a minimum of 169 students were needed for the sample to be representative.

### 2.3. Conceptualization

To create the virtual patient that we called Julia, we chose to create a conversational chatbot with AI. To this end, a working group was created with two professors of dentistry from the Universidad Europea de Madrid to begin the conceptualization work and define everything that was necessary for Julia to present as a patient. In this study, it was decided that she suffered from reversible pulpitis. 

**Figure 2:**
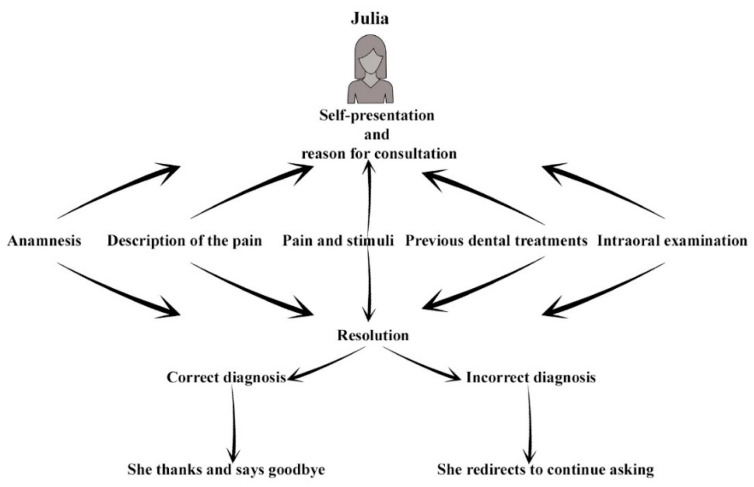
Chatbot conceptualization diagram.

After an analysis of the literature [33,34], five main categories were defined for her to answer: anamnesis, description of the pain, relationship of the pain with stimuli, previous dental treatments, and intraoral exploration. In order to establish a dose of reality and to create more interest among the students, it was decided to create the chatbot using an informal language that could answer some questions that were unrelated to the clinical case. Figure 2.

Subsequently, work was done to create sub-categories in which the most frequently used expressions were included with more informal linguistic variations to which a response was associated in order to establish a flow of dialogue (Table 1).

### 2.4. Chatbot Design

The Dialogflow^®^ application (Palo Alto, Santa Clara County, CA, USA), was used for the creation of chatbot conversational flows through the use of intuitive artificial intelligence [35] that was capable of understanding the nuances of human language by learning through action and feedback.

**Figure 3:**
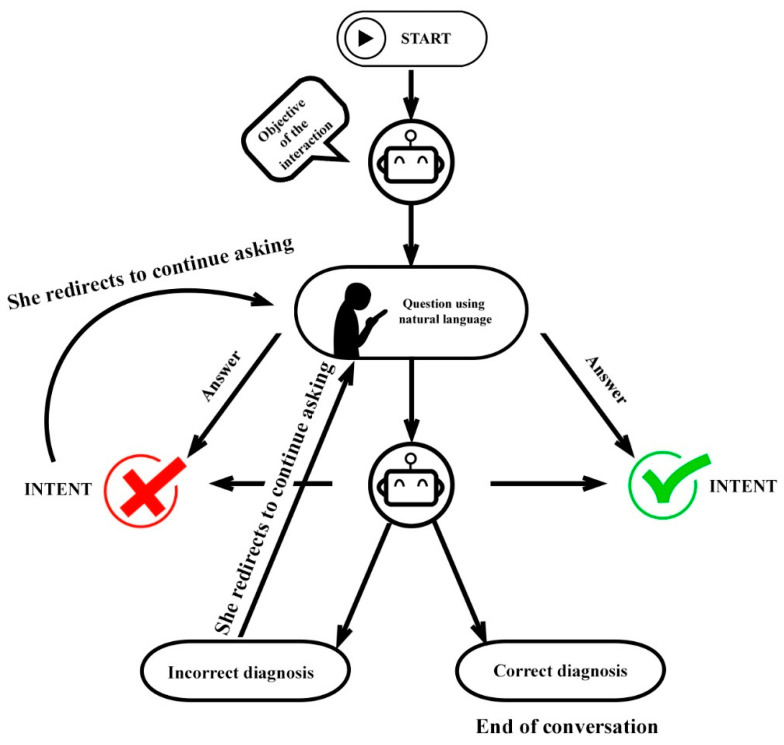
Data flow diagram.

Since the people who created the chatbot were not experts in the field, it was decided to design the chatbot in a simple way. To do this, we defined the “intents” (or what the user wanted to say), added all the expressions that a user could use to express that “intent” and that the group of experts had defined in the previous phase to add them in the “training phrases” space, and then associated a specific response to that intent. Through natural language processing algorithms, the AI will be able, with a few training phrases, to learn the different ways of asking the same question (Table 2 and Figure 3).

Once the chatbot was created, it was integrated with an instant messaging application (Telegram) because it was intended to offer this experience easily, quickly, and using an application that was frequently used by students, also giving them the possibility of interacting with Julia at any time. 

In order to carry out the integration of Julia in Telegram, the application was accessed and then the following steps were followed:

1. Go to https://telegram.me/botfather (accessed on 19 April 2022)
2. Type/start
3. Type/newbot
4. Create a name ending in “bot”.
5. Then Telegram generates a token to access the hhtp API.
6. In Dialogflow, go to “Integrations” and then click on the Telegram icon.
7. Paste the token in the corresponding field and click on “start”.

**Figure 4:**
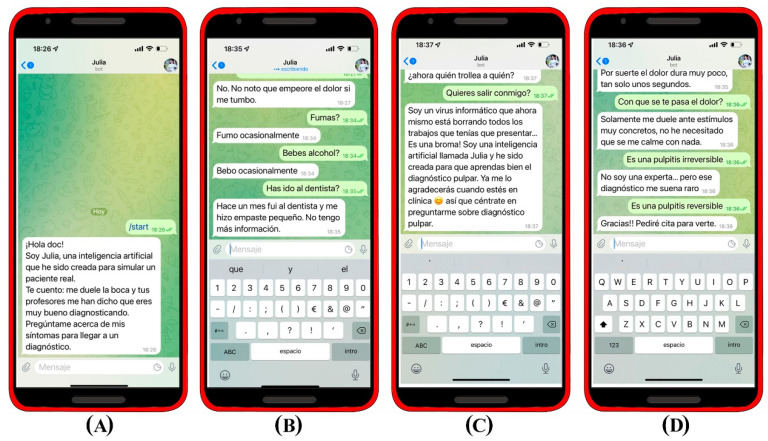
Example of a conversation flow. (**A**) At the beginning of the interaction with Julia, she introduces herself and makes directions about what the student should do. (**B**) Julia is able to answer different questions about the current condition. (**C**) Colloquial responses to intimate questions that were unrelated to the case were established in order to arouse students’ curiosity and redirect them. (**D**) In case of reaching an incorrect diagnosis, Julia redirects the student.

In order for Julia to generate curiosity among the students and given the possibility that some questions were not focused on the clinical case, “intents” were created for various questions such a *“Do you want to go out with me?”* generating natural answers that would lead the student back to the main objective of the chatbot, the pulp diagnosis: *“I’m a computer virus that right now is deleting all the papers you had to submit…it’s a joke! I’m an artificial intelligence named Julia and I’ve been created for you to learn pulp diagnosis well. You will thank me when you are in the clinic. So focus well and ask me about pulpal diagnosis”*. When students gave an incorrect diagnosis, Julia encouraged them to keep asking *“I’m not an expert… but that diagnosis sounds weird to me”* in case of giving correct answer Julia replies and closes the chat *“Thank you! I will make an appointment to see you”.* Figure 4.

### 2.5. Start-Up

**Figure 5:**
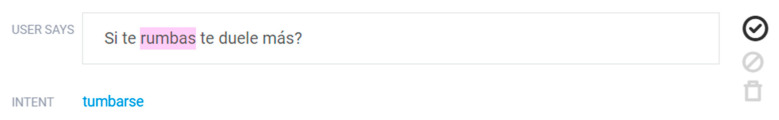
If the user misspelled a word and the AI was able to identify that it was an error and associate it with the correct intent.

The operationalization was carried out in two phases. In the first instance, a panel of experts consisting of 5 professors and doctors of dentistry interacted with Julia. All of the failed interactions or evidenced errors were reported for further adjustment to improve the chatbot conversation flow. For this purpose, the Dialogflow training function was used to test those interactions with users that the AI itself considers should be revised. In this way, the AI is learning from the actions that it performs and the feedback we give it (Figure 5).

When the validation by expert judgment was positive, we proceeded to a second phase in which Julia was sent to 4th and 5th year dental students with all the information and the route to interact with Julia via Telegram.

### 2.6. Survey

After four weeks of operation, the students who were interested in participating in the study were asked to fill out an eleven-question questionnaire in which nine questions dealt with their experience after their interaction with Julia and two open-ended questions (Table 3 and Table 4).

### 2.7. Statistical Análisis

The questionnaire responses were collected and the data were entered into a Microsoft Excel spreadsheet. They were then analyzed using SPSS software (IBM, SPSS Statistics, Version 20.0, Armonk, NY, USA: IBM Corp). 

The Kolgomorov–Smirnov test was performed to evaluate whether the samples met the normality criterion. For comparisons between the courses and sex, the Student’s *t*-test was used for those samples that had a normal distribution and the Mann–Whitney U test for those that did not; for the association between the qualitative variables, the chi-square test was used, considering the *p*-value ≤ 0.05 as statistically significant.

## 3. Results

The sample size of the study was 193 subjects, of whom 58 belonged to the fourth year and 135 to the fifth year. There were 109 females and 84 males. In fourth year, women accounted for 55.2% and men 44.8% of the sample while in fifth year, women accounted for 57.04% and men 42.26%.

### 3.1. Global Data

**Figure 6:**
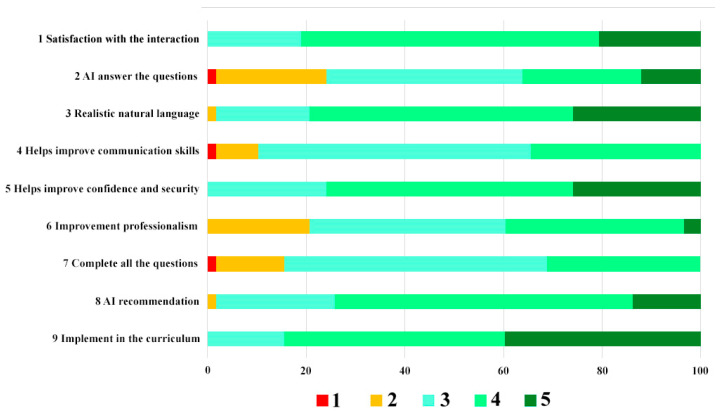
Distribution of responses per questionnaire item by fourth year dental students. 1-Strongly Disagree, 2-Disagree, 3-Neutral, 4-Agree, 5-Strongly Agree.

**Figure 7:**
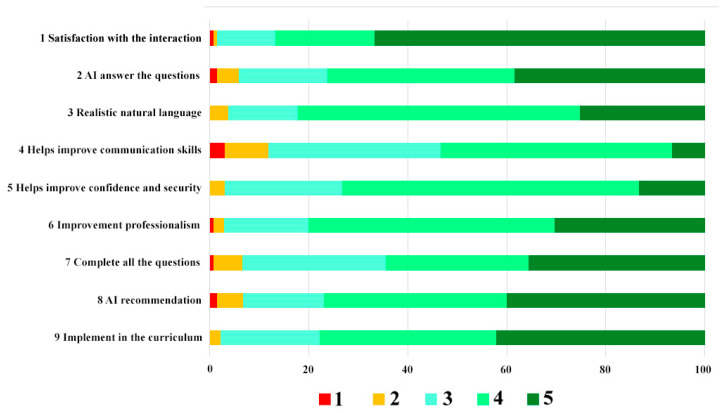
Distribution of responses per questionnaire item by fifth year dental students. 1-Strongly Disagree, 2-Disagree, 3-Neutral, 4-Agree, 5-Strongly Agree.

The results of the response to the questionnaire, which were measured with a Likert scale (1–5), are shown in Table 5 and in Figure 6 and Figure 7.

When comparing the responses to the questionnaire by course, statistically significant differences were found, with fifth-year students showing the highest satisfaction values (Table 6 and Table 7).

In relation to gender, the *t*-test found that women rated the realistic natural language of AI better (*p-value = 0.008*).

When the Chi-square test (χ^2^) was performed, the results showed that the fifth year students got the diagnosis right more frequently (*p-value = 0.005*) than the fourth year students. When comparing between sexes, females failed more often than males (*p-value = 0.000*).

We also looked for whether there was a correlation between establishing a correct diagnosis and a higher score on the questionnaire. When the Chi-square (χ^2^) test was performed, it was observed that a correct diagnosis implied a higher score on the questionnaire items (Table 8).

In the second free field of the questionnaire, the students were asked about what could be modified or added to the AI. The responses are shown in Table 9.

### 3.2. Fourth Year Student Data

When the Mann-Whitney U test was used to compare the values of each of the responses to the questionnaire items with sex, no significant differences were obtained. When the χ^2^ test was performed to compare the correct diagnosis with sex, no significant differences were obtained.

### 3.3. Fifth Year Student Data

When comparing the values of the items with sex through the Mann–Whitney U-test, statistically significant values were obtained in the item “realistic natural language” (*p* = *0.022*), with women scoring higher, and in the item “complete all the questions” (*p* = *0.042*), with men scoring higher. When χ^2^ was performed to compare the correct diagnosis vs. sex, 19.26% of women failed more than men with 5.19% (*p* = *0.004*).

## 4. Discussion

The university must respond to the dynamic needs of current technological updating. In this sense, AI presents itself as a novel and unfamiliar resource for many trainers, but it has the potential to achieve effective learning [36,37]. In fact, it is claimed that students can improve their skills and knowledge if, in addition to interacting with human teachers, they interact with technological trainers who have reasoning and decision-making capabilities that are similar to human ones [6,36,38,39].

AI has experienced great advances in recent years, causing a great impact on science, economics, and education [36]. In reference to the field of education, in some previous studies with students of health branches [40,41], they valued very positively, as in the present study, the interaction with artificial intelligences. Moreover, as in this study, they affirmed the need to implement this technology in the curricula. However, AI also presents certain limitations. It has been shown that a possible limitation would be related to the knowledge about artificial intelligence and machine learning of students [42]. In addition, it has been observed that some students may be reluctant to accept these technological developments as they consider that they have greater learning with a teacher interacting face-to-face and not on-line, being interaction and error correction one of the basic learning points for them [43,44]. Moreover, students who teach with patients highly value observational or vicarious learning [45] together with their fellow trainees. All of these reasons may explain the lack of updating in these developments in dental school [40].

Any simulation-based learning should be based on sound principles of prior knowledge [46], so this study was conducted with final-year dental students treating real patients, as there is an integration of theory with practice. In addition, students often present difficulties in diagnostic competence and VPs offer more practical opportunities to improve their future performance with patients [6]. This may be the reason why discrepancies between diagnostic successes are observed, with final year students scoring clearly higher than fourth year students.

With real patients, situations are very changeable, so varying degrees of difficulty, and these situations can be counterproductive for students due to the frustration and distress they may be subjected to [8]; in this sense, VPs can recreate in a controlled, stimulating, and safe environment, the doctor-patient relationship [47] and encourage reflective learning [6,41].

In the dental students’ interaction with the virtual patient Julia, we focused on the ability to obtain a preliminary diagnosis with the data provided in a direct conversation because the collection of information during the patient interview significantly influences the quality of the diagnosis [48]. As the preliminary diagnosis must be confirmed with complementary tests [49], Julia requested a subsequent appointment at a clinic when the diagnosis was correct.

In relation to the development budget, the economic view of this technological resource cannot be ruled out since it has been shown that virtual simulation minimizes the cost of the activity compared to simulation that is based on traditional simulators (mannequins), high-fidelity simulators, haptic simulators, as well as the use of standardized patients (actors) [6,11]. In the present study, the high economic investment that is traditionally also associated with innovative developments was ruled out, since it was possible to recreate a VP using the free version of a very intuitive software. In order to carry out the step-by-step creation of Julia and its integration into the instant messaging program, the indications of the numerous free tutorials that are available online were followed.

During the testing phases and in the first days of operation, it was observed that not being able to identify users increased the risk of asking controversial questions, off-target questions to make Julia feel bad, or simply questions that were asked to observe the possible reaction of the artificial intelligence. Due to this, a collection of insults, rude phrases, out-of-place comments, etc. was also carried out in order to redirect the users. During the implementation, it was possible to see how a small group of users tried to “troll” Julia and how she redirected the user to the activity using a sarcastic text. 

The fifth year students showed greater satisfaction in all the items of the questionnaire, perhaps due to their almost two years of practice on patients and the global vision of curricular development that can be perceived when graduation is near. In addition, in the free text field, they were the ones who expressed greater satisfaction with the interaction and proposed the possibility of implementing this technology in pre-clinical courses. On the contrary, the fourth year students rated the interaction with Julia worse, being more critical with the difficulty of the case, with the language that was used, and they also needed the possibility that the patient could answer several questions at the same time, etc.

All the data that were collected in the study lead us to think that VPs through chatbot with AI should be adapted to each course and type of student. In the case of fourth year students, who are beginning to have contact with real patients, perhaps it should be more oriented towards practice and the development of anamnesis skills during medical history taking so that they could practice more times and thus feel more confident with their first patients. On the contrary for fifth year students, more complex and challenging scenarios should be developed by providing complementary material such as radiographs, laboratory tests, photographs, etc. Authors such as Joda et al. [50] also propose increasing the realism of VPs with avatars in which skin and tissues are replicated by superimposing and merging 3D images, these lines of research continue to be developed and it is hoped that, in the near future, it will be part of the curriculum for dental students as a complement to face-to-face interaction with patients. In relation to this last point, we should emphasize the importance in dental practice of the dentist’s empathy, the ability to recognize nonverbal communication, establish bonds of trust with patients, know their expectations and fears, etc. [21], feelings that today no machine can replicate as they are exclusive to human beings [51].

## 5. Conclusions

Our results highlight the usefulness of simulating a VP with AI by giving students the possibility of multiple clinical cases to practice, as well as offering an engaging and personal experience to students because of the interface and the natural language that are used, without underestimating the economic and space savings for universities. Therefore, our research suggests the need to incorporate AI into dental curricula while also ensuring that students are at the forefront of current technological developments.

## Figures and Tables

**Table 1:** Example of the expressions for a question about Julia’s response if heat is applied with a corresponding answer.

Sub-Categories	Expressions	Answer
Heat	Do you have discomfort in the heat? Do you have pain in the heat? Are you sensitive to heat? Are you bothered by hot things? Does it bother you with high temperature? Does it hurt with high temperatures? Does it hurt with high thermal stimulation? Does it hurt with high temperature? Does it hurt if you eat something hot? Does it hurt if you drink something hot?	No, with the heat I don’t feel any pain.

**Table 2:** Question-answer sequence of the chatbot.

Intents	Training Phrases	Answer
Cold (pulp response to cold application)	Does it hurt to drink something refrigerated? Do you have pain with something cold? Do you feel more sensitive when you drink something cold? Do you feel more sensitivity when eating cold things? Does it hurt to drink something cool? Does it hurt if you drink something with ice? Does it hurt more with cold? If you drink something cold, do you feel it?	Yes, when I drink something cold I feel pain

**Table 3:** Questions of the questionnaire with possible answers.

Questions	Possible Answers (Only One)
1-Were you satisfied when interacting with the artificial intelligence? 2-Did the artificial intelligence answer all your questions about the pulp pathology I presented? 3-Did the language used by the artificial intelligence seem natural and realistic to you? 4-Do you feel that this type of teaching methodology can help you improve your communication skills? 5-Do you think this type of teaching methodology can help you feel more confident and secure when treating patients? 6-Do you think that this type of teaching methodologies could help you grow as a future professional? 7-Did you manage to ask all the necessary questions to reach a pulp diagnosis? 8-Would you recommend this artificial intelligence-based technology to other students? 9-Do you think that interaction with artificial intelligences should be part of the dental degree curriculum?	1-Strongly Disagree 2-Disagree 3-Neutral 4-Agree 5-Strongly Agree

**Table 4:** Free text response questions.

Open Questions
-What pulp pathology do you think the patient had? -What would you modify or add after interacting with this artificial intelligence.

**Table 5:** Descriptive statistics by item.

	4th		5th		Total	
	Mean	S. d.	Mean	S. d.	Mean	S. d.
1 Satisfaction with the interaction	4.02	0.083	4.51	0.068	4.36	0.056
2 AI answers the questions	3.22	0.130	4.07	0.081	3.82	0.074
3 Realistic natural language	4.03	0.095	4.04	0.063	4.04	0.053
4 Helps improve communication skills	3.22	0.089	3.45	0.074	3.38	0.059
5 Helps improve confidence and security	4.02	0.094	3.84	0.059	3.89	0.050
6 Improvement professionalism	3.22	0.107	4.07	0.068	3.81	0.064
7 Complete all the questions	3.14	0.093	3.93	0.084	3.69	0.070
8 AI Recommendation	3.86	0.087	4.09	0.082	4.02	0.063
9 Implement in the curriculum	4.24	0.093	4.18	0.071	4.20	0.057

S. d.: standard deviation.

**Table 6:** Mann–Whitney U-test results.

	Sig. (Bilateral)
1 Satisfaction with the interaction	0.000 ** ^5th^
2 AI answers the questions	0.000 ** ^5th^
6 Improvement professionalism	0.000 ** ^5th^
7 Complete all the questions	0.000 ** ^5th^
8 AI Recommendation	0.016 * ^5th^

* *p*-*value < 0.05*—statistically significant. ** *p-value < 0.001*—highly statistically significant. 5th = 5th > 4th.

**Table 7:** Student’s *t*-test results.

	d.f.	Sig. (Bilateral)
3 Realistic natural language	191	0.982
4 Helps improve communication skills	136.002	0.051
5 Helps improve confidence and security	191	0.099
9 Implement in the curriculum	191	0.610

d.f.: degrees of freedom.

**Table 8:** χ^2^ test. Diagnosis vs. questionnaire.

	Value	d.f.	Asymptotic Significance (Bilateral)
1 Satisfaction with the interaction	9.496	4	0.050 *
2 AI answers the questions	23.992	4	0.000 **
3 Realistic natural language	11.647	3	0.009 *
4 Helps improve communication skills	22.166	4	0.000 **
5 Helps improve confidence and security	8.899	3	0.031 *
6 Improvement professionalism	24.636	4	0.000 **
7 Complete all the questions	97.764	4	0.000 **
8 AI Recommendation	14.320	4	0.006 *
9 Implement in the curriculum	13.362	3	0.004 *

d.f.: degrees of freedom. * *p-value < 0.05*—statistically significant ** *p-value < 0.001*—highly statistically significant.

**Table 9:** Responses to the free text field in which students could add their impressions after the interaction.

The colloquial language should be expanded. It should answer several questions at the same time. The language is very complete but does not always respond to colloquial phrases. Lack of feedback, although being like a real patient it is logical that you do not get it. Very curious. Very interesting. It would have been nice to see it in pre-clinical courses. Should not replace a patient. Cannot establish the diagnosis because the patient did not define time of pain. duration in the cold sensitivity test. Does not resemble a patient. Should have the possibility to add images. We should be able to make an appointment. I would like to get the right answer. I would want an option to know the correct diagnosis after mine. X-rays. You could have many to practice. Super interesting to practice. A simpler patient.

## Data Availability

Not applicable.
